# Identification of *Vibrio parahaemolyticus* and *Vibrio* spp. Specific Outer Membrane Proteins by Reverse Vaccinology and Surface Proteome

**DOI:** 10.3389/fmicb.2020.625315

**Published:** 2021-01-28

**Authors:** Wenbin Wang, Jianxin Liu, Shanshan Guo, Lei Liu, Qianyun Yuan, Lei Guo, Saikun Pan

**Affiliations:** ^1^Jiangsu Key Laboratory of Marine Biotechnology, Jiangsu Ocean University, Lianyungang, China; ^2^Co-Innovation Center of Jiangsu Marine Bio-industry Technology, Jiangsu Ocean University, Lianyungang, China; ^3^Jiangsu Key Laboratory of Marine Bioresources and Environment, Jiangsu Ocean University, Lianyungang, China

**Keywords:** *Vibrio parahaemolyticus*, outer membrane protein, identification, surface proteome, bioinformatics, shaving, reverse vaccinology

## Abstract

The discovery of outer membrane proteins (OMPs) with desirable specificity and surface availability is a fundamental challenge to develop accurate immunodiagnostic assay and multivalent vaccine of pathogenic *Vibrio* species in food and aquaculture. Herein 101 OMPs were systemically screened from 4,831 non-redundant proteins of *Vibrio parahaemolyticus* by bioinformatical predication of signaling peptides, transmembrane (TM) α-helix, and subcellular location. The sequence homology analysis with 32 species of *Vibrio* spp. and all the non-*Vibrio* strains revealed that 15 OMPs were conserved in at least 23 *Vibrio* species, including BamA (VP2310), GspD (VP0133), Tolc (VP0425), OmpK (VP2362), OmpW (VPA0096), LptD (VP0339), Pal (VP1061), flagellar L-ring protein (VP0782), flagellar protein MotY (VP2111), hypothetical protein (VP1713), fimbrial assembly protein (VP2746), VacJ lipoprotein (VP2214), agglutination protein (VP1634), and lipoprotein (VP1267), Chitobiase (VP0755); high adhesion probability of flgH, LptD, OmpK, and OmpW indicated they were potential multivalent *Vibrio* vaccine candidates. *V. parahaemolyticus* OMPs were found to share high homology with at least one or two *Vibrio* species, 19 OMPs including OmpA like protein (VPA073), CsuD (VPA1504), and MtrC (VP1220) were found relatively specific to *V. parahaemolyticus*. The surface proteomic study by enzymatical shaving the cells showed the capsular polysaccharides most likely limited the protease action, while the glycosidases improved the availability of OMPs to trypsin. The OmpA (VPA1186, VPA0248, VP0764), Omp (VPA0166), OmpU (VP2467), BamA (VP2310), TolC (VP0425), GspD (VP0133), OmpK (VP2362), lpp (VPA1469), Pal (VP1061), agglutination protein (VP1634), and putative iron (III) compound receptor (VPA1435) have better availability on the cell surface.

## Highlights

- OMPs between *Vibrio species* had a close and complex genetic relationship.
- The capsule affected surface proteome analysis of *V. parahaemolyticus.*
- Differential OMPs of *V. parahaemolyticus* and *Vibrio* spp. were revealed.

## Introduction

*Vibrio* spp. are halophilic bacteria that are widely distributed in seawater, offshore sediments, marine life, and seafood products (Hackbusch et al., 2020). Due to the high prevalence of *Vibrio* species in seafood, *Vibrio*-related food poisoning represents the leading cause of infectious diarrhea in coastal regions (Elmahdi et al., 2016). Acute attacks, abdominal pain, and watery symptoms are the main clinical symptoms (Guin et al., 2019). The most prevalent pathogenic *Vibrio* species in human infections include *Vibrio parahaemolyticus*, *Vibrio vulnificus*, and *Vibrio cholera*, among which *V. vulnificus* is more life threatening and *V. parahaemolyticus* exhibits a higher prevalence in sea food (Bonnin-Jusserand et al., 2019). Furthermore, the vibriosis caused by *Vibrio* species (e.g., *V. parahaemolyticus*, *Vibrio anguillarum*, *Vibrio alginolyticus*, *Vibrio ordalii*, and *Vibrio harveyi*) has been found to cause an infection in more than 50 economic fish species and is considered a major economic threat to the marine aquaculture industry (Yu et al., 2016).

Rapid diagnostic tests are promising to provide better surveillance in the aquaculture and food industries (Baker-Austin et al., 2018). Although there have been several reports on the preparation of diagnostic antibodies against *V. parahaemolyticus* or *Vibrio* species by whole cell antigens, flagella, and hemolysins, the specific diagnostic surface antigens are largely unknown (Xu et al., 2019). Also, vaccines are the preferential way to battle vibriosis in aquaculture for sustainability and food safety (Wang et al., 2020). Polyvalent vaccines that cover the main serotypes of the pathogen are preferred when compared with the monovalent vaccine that contains a single strain of a single antigen (Schlingmann et al., 2018). Furthermore, the multiple vaccine (combination vaccine) combined two or more vaccines have effectively protected groupers against multiple *Vibrio* and viral pathogens (Huang et al., 2012). The traditional strategy of *Vibrio* polyvalent vaccines is based on the whole-cell antigens of representative strains like *V. alginolyticus*, *V. parahaemolyticus* (Aly et al., 2020). The protection against homologous strains with these vaccines was very effective, but the protection of heterogeneous strains or multiple species were elusive. Outer membrane proteins (OMPs) are currently the main candidate antigens used in polyvalent vaccine studies for their essential function, surface exposure, and conservation among different strains (Pore and Chakrabarti, 2013). There have been some studies of the OmpK and OmpU as polyvalent vaccine candidates (Duperthuy et al., 2010; Li et al., 2010b), while many studies focus on the discovery of more multivalent and potent Omps for future vaccine development.

Based on the bacterial serum-based immunoproteomics, Li et al. (2010a) identified 33 OMPs of *V. parahaemolyticus* on the two-dimensional (2-DE) gel, and found that the OmpA and Pal proteins had a multiple protective effect for the tested strains of *Vibrio*, *Aeromonas*, and *Pseudomonas* in carp. They also revealed the polyvalent vaccines from *V. alginolyticus* by a heterogeneous antiserum-based immunoproteomics with bacterial immunization challenging method (Li et al., 2009). Li et al. (2014) in another group used the same strategy and found 10 immunogenic OMPs, including LptD, and OmpK. Comparative analysis of Omps that respond to fish and human plasma stress by differential sub-proteomic methodologies was also used to develop highly protective vaccine candidates of *E. tarda* (Wang et al., 2012). DNA shuffling of six OmpA gene from *V. alginolyticus*, *V. parahaemolyticus*, *Edwardsiella tarda*, and *Escherichia coli* was also reported to develop polyvalent vaccines against *V. alginolyticus* and *E. tarda* infections (Cheng et al., 2018). However, the multivalence of these proteins among the *Vibrio* species remains unclear and some conserved OMPs with a lower abundance may be missed due to competition of the immune system for whole-cell immunogen (Shinoy et al., 2013). Another proteomics strategy is the enzymatic shaving-based surface proteome analysis (Rodríguez-Ortega et al., 2006). It was successfully used to identify the surface-exposed proteins of Gram-positive group A *Streptococcus*, and widely applied to some Gram-negative bacteria (Solis and Cordwell, 2011; Olaya-Abril et al., 2014). No shaving-based surface proteome study of *Vibrio* has been reported.

A genomic and bioinformatic approach, also known as reverse vaccinology, has revolutionized antigen screening and vaccine development by adopting computerized screening of all protein sequences from the target pathogen (Dalsass et al., 2019). Pizza et al. (2000) first reported an analysis of the open reading frames of *Neisseria meningitidis* and intensive recombinant expression of the 570 potential OMPs (Giuliani et al., 2006). To improve the labor-intensive screening process, many bioinformatics tools have been used to analyze large quantities of genomic or protein sequences (Garcia-Angulo et al., 2014). Specially, to reveal broad-spectrum immunogenic targets and analyze the close-related species, the pangenome analysis for the core, accessory, and unique genes was introduced to reverse vaccinology (Zeng et al., 2017; Naz et al., 2019). To our knowledge, the OMPs of *V. anguillarum* have been screened by reverse vaccinology (Baliga et al., 2018). However, little is known about the immunogenic OMPs that are specific to *V. parahaemolyticus* or *Vibrio* spp. and represent promising diagnostic antigens and vaccine candidates.

In this study, the OMPs of *V. parahaemolyticus* were comprehensively screened from the 4,831 protein sequences with intergraded bioinformatics predication methods. The differential OMPs and vaccine candidates of *V. parahaemolyticus* and *Vibrio* spp. were obtained after the overall homology analysis with the other bacteria using the Basic local alignment search tool (BLAST,^1^). The possible vaccine candidates were further selected by the adhesion probability analysis. To experimentally study the OMPs of *V. parahaemolyticus*, the surface proteome was analyzed with enzymatical shaving (glycosidases, trypsin) of the intact cells and mass spectrometry identification of the related peptides.

## Materials and Methods

### Screening of Signal Peptide-Positive Proteins From the Encoded Proteins of *V. parahaemolyticus* in the National Center for Biotechnology Information

**FIGURE 1:**
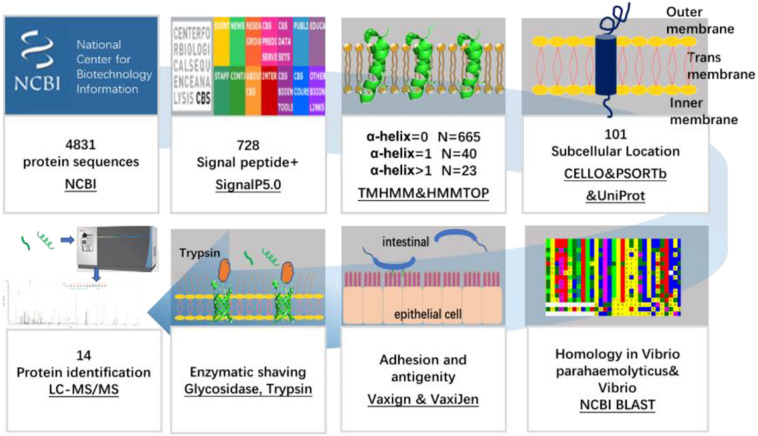
Schematic diagram of a bioinformatical and proteomic approach for the identification of OMPs against *V. parahaemolyticus* and *Vibrio* spp.

The flow chart of OMP screening of *V. parahaemolyticus* is shown in Figure 1.

The 4,831 protein sequences encoded by nucleotides (accession numbers: BA000031.2 and BA000032.2) of the *V. parahaemolyticus* RIMD 2210633 strain were downloaded as FASTA files from the National Center for Biotechnology Information (NCBI) database. The signal peptide is involved in protein transportation and is located on the cell membrane. All of the 4,831 encoded proteins of *V. parahaemolyticus* were analyzed for the presence of the N-terminal signal peptide using SignalP 5.0 (Armenteros et al., 2019). The following parameters were used, and the maximum number of inputted proteins was 5,000. The organism group was Gram-negative and the output format was a short output.

### Predicting the Transmembrane Helices in the Candidate Proteins

The number of highly hydrophobic TM helices in the proteins was negatively associated with the possibility of recombinant expression. The topology information revealed the location of aminol acids on the protein structure and the cell membrane. The transmembrane (TM) helix and topology of the candidate proteins were predicted using the online server TMHMM 2.0 (Erik et al., 1998) and HMMTOP (also known as Community College Transfer Opportunity Program [CCTOP]) (Tusnády and Simon, 2001). Due to the substantial challenge of multi-pass TM protein expression in the prokaryotic expression system, the proteins with less than two TM helixes were selected for further analysis.

### Predicting the Subcellular Localization of the Candidate Proteins

The bacterial proteins are primarily located in the cytoplasm, the inner membrane (cytoplasmic membrane), periplasm, outer membrane, and extracellularly. The subcellular locations of the obtained proteins with less than two TM helixes were predicted by PSORTb, CELLO, and UniProt. PSORTb is a web-based tool that provides a link to the PSORT family of programs for predicting the subcellular localization (Yu et al., 2010). CELLO based on Support Vector Machine was used to detect specific amino acid compositions and motifs, thereby predicting the subcellular location of proteins (Yu et al., 2004). The UniProt database provides a comprehensive, high-quality, and freely accessible resource of protein information, including the location and topology of the mature proteins in the cell (The UniProt, 2018). To cross-check the results and improve the coverage and accuracy of prediction, proteins predicted as the OMP by more than one method were selected for further analysis.

### Sequence Homology Analysis of the Candidate Proteins With Large Number of Strains

Basic local alignment search tool is an online tool used to identify the local similarity between sequences. This program is supported by the NCBI to compare the nucleotide or protein sequences with sequence databases and calculate the statistical significance of matches. BLAST can also be used to infer the functional and evolutionary relationships between sequences and help identify the protein families. The BLAST program was used to determine the homology with all the identified OMPs among *V. parahaemolyticus* serovars, as well as those present in the other 32 *Vibrio* spp. (*Vibrio cholerae*; *V. vulnificus*; *V. alginolyticus*; *Vibrio diabolicus*; *Vibrio antiquarius*; *Vibrio campbelli*; *V. harveyi*; *Vibrio owensii*; *Vibrio rotiferianus*; *Vibrio jasicida*; *Vibrio natriegens*; *V. alfacsensis*; *V. coralliilyticus*; *V. anguillarum*; *Vibrio splendidus*; *Vibrio mimicus*; *Vibrio cincinnatiensis*; *Vibrio metschnikovii*; *Vibrio gazogenes*; *Vibrio mediterranei*; *Vibrio diazotrophicus*; *Vibrio fluvialis*; *Vibrio furnissii*; *Vibrio proteolyticus*; *Vibrio nereis*; *Vibrio tubiashii*; *Vibrio nigripulchritudo*; *Vibrio aerogenes*; *Vibrio orientalis*; *V. ordalii*; *Vibrio aestuarianus*; and *Vibrio carchariae*), seven other marine bacteria (*Aliivibrio*; *Salinivibrio*; *Enterovibrio*; *Grimontia*; *Photobacterium*; *Plesiomonas*; and *Shewanella*), and six common bacteria (*E. coli*; *Aeromonas*; *Salmonella*; *Pseudomonas*; *Acinetobacter*; and *Yersinia*). The seven marine bacteria were selected because they were found to exhibit a relatively high sequence homology with the candidate proteins in an overall BLAST with non-*Vibrio* spp. strains.

### Antigenicity and Adhesion Index Analysis of the Candidate Proteins

Antigenicity refers to the ability of an antigen to be recognized by the immune system. The potential antigenicity of the candidates was estimated through VaxiJen (Doytchinova and Flower, 2007) with a cut-off of 0.4. Adhesion-related OMPs are involved in the pathogenicity of the bacteria and have a greater priority for vaccine development. Vaxign (He et al., 2010) predicts the possible vaccine candidates according to various vaccine design criteria (e.g., sub-cellular location, adhesion, and trans-membrane helix). The predication of Vaxign programs has corresponding sensitivity and specificity values of 0.494 and 0.853 (Jaiswal et al., 2013). Proteins with a Vaxign index higher than 0.5 were predicted to be adhesion-related proteins and selected as vaccine candidates.

### Surface Proteome of *V. parahaemolyticus* Analyzed by Trypsin Shaving and Mass Spectrometry

The surface proteome of *V. parahaemolyticus* was studied by “shaving” the cells with trypsin as described (Rodríguez-Ortega, 2018; Hornburg et al., 2019). Briefly, *V. parahaemolyticus* (CICC 21617) was cultured in Luria-Bertani broth with 3.0% NaCl at 37°C until the OD_600_ was 0.38, which corresponded to the mid-exponential phase. The culture (50 mL) was centrifuged at 3,500 g, 10 min, 4°C. The pellet was gently suspended in 5 mL 1% paraformaldehyde (PFA) (Aladdin Bio-Chem Technology, Shanghai, China) in PBS for 5 min and washed twice with PBS. One set of the sample was resuspended in 1 mL shaving buffer (1× PBS, 20% [w/v] sucrose, 10 mM DTT) and added with 20 μg sequencing grade trypsin (Sigma-Aldrich, St. Louis, MO, United States) in 0.5 mL 25 mM ammonium bicarbonate buffer. The treated samples were incubated at 37°C in water bath for 15 and 30 min. The control was added with the 0.5 mL 25 mM ammonium bicarbonate buffer and incubated at 37°C in water bath for 30 min. In a further study, the cells were first treated with endoglycosidase α-amylase (Macklin Biochemical Co., Ltd., Shanghai, China), cellulase (solarbio, Beijing, China), pullulanase (Shijiazhuang Ningnuo Trading Co., Ltd., Hebei, China), dextranase (G-CLONE, Biotech Co., Ltd., Beijing, China), and exoglycosidase starch glucosidase (Macklin Biochemical Co., Ltd., Shanghai, China) to reduce the capsule and lipopolysaccharide on the cell surface before “shaving” with trypsin. The cell pellets of 50 mL culture were resuspended in 5 mL phosphate buffer (pH 5.5) with final concentration of 100 U/mL of α-amylase (α-1, 4-linkage), cellulase (β-1, 4), pullulanase (α-1, 6), dextranase (β-1, 3; β-1, 4), and starch glucosidase (α-1, 4; α-1, 6; and α-1, 3). The samples were incubated at 50°C in water bath for 6 h. The content of saccharides in the supernatant was measured by phenol sulfuric acid method (Cuesta et al., 2003). Then the cells were centrifuged, washed by PBS, and treated with the trypsin by the same way as described above. After centrifugation at 8,000 g, 10 min, 4°C, the samples were filtered with a 0.22-μm pore-size filter and sent to Sangon Biotech (Shanghai) Co., Ltd. for the 30 min LC-MS/MS analysis. The MS/MS data was acquired when the precursor ion signal was greater than 120° and the charge number was +2∼+5. The database used for searching was the proteome reference database of *V. parahaemolyticus O3:K6 (strain RIMD 2210633)* in UniProt. Two trial of the experiments were conducted and three replicates of each sample were tested.

### Structure Simulation of the Candidate Proteins

The three-dimensional protein structure was simulated by the online servers, I-TASSER (Yang et al., 2015), and TrRosetta (Yang et al., 2020). I-TASSER is an iterative template-based fragment assembly simulation server. TrRosetta builds the protein structure based on direct energy minimization with a restrained Rosetta. Briefly, the sequence of the candidate protein was inputted and submitted to the server with an automatic model, and a high-quality simulated protein structure is obtained when the C-score is more than −1.5 in I-TASSER, the C-score is greater than −1.5, and a Tm score more than 0.5 in TrRosetta (Xu and Zhang, 2010).

## Results

### Bioinformatical Identification of *V. parahaemolyticus* Outer Membrane Proteins

In this study, screening of the 4,831 proteins encoded by *V. parahaemolyticus* RIMD 2210633 revealed 728 proteins that had a signal peptide sequence in the N-terminal region. Among them, 505 were general secretory pathway (Sec) signal peptides, 21 were twin-arginine translocation (TAT) signal peptides, and 202 were lipoprotein signal peptides. The outer membrane proteome of bacteria is dominated by a β-barrel with no TM α-helix or α-helical membrane proteins with one or more TM α-helix (Rollauer et al., 2015). TMHMM 2.0 and HMMTOP showed 665 proteins had no TM α-helix and 40 proteins had one TM α-helix, which indicated they were primarily β-barrel proteins. The β-barrel proteins are mainly porin proteins that occur only in the outer membranes of bacteria and are involved in metabolite and protein transport, osmotic pressure regulation, and numerous signaling processes (Wimley, 2003). The other 23 proteins were excluded for further analysis due to a substantial challenge of multi-pass TM protein expression in a prokaryotic expression system as reported by Serruto (Serruto et al., 2004).

**FIGURE 2:**
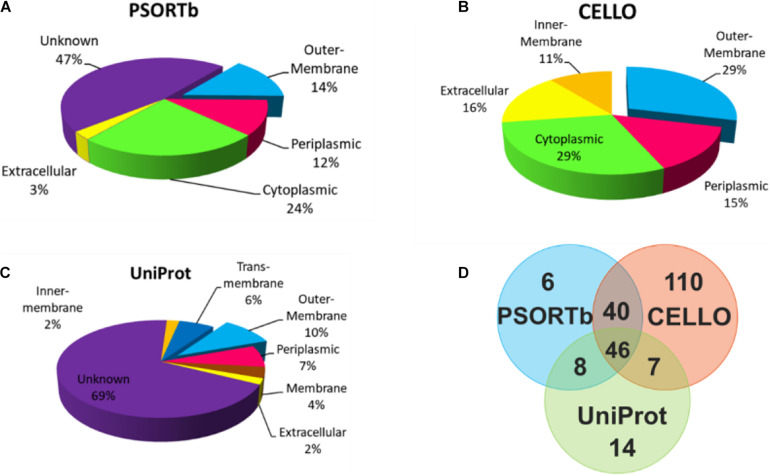
Subcellular location analysis of the 705 proteins by PSORTb **(A)**, CELLO **(B)**, and UniProt **(C)**. **(D)** Mapping the OMPs predicted by three methods.

To select OMPs that are primarily involved in the host environment interaction and exclude the cytoplasmic proteins (Sanober et al., 2017), the subcellular localization of the proteins was predicted using three parallel online approaches. Of the 705 *V. parahaemolyticus* proteins, PSORTb, CELLO, and UniProt revealed that 100 (14.2%), 203 (28.8%), and 75 (10.6%), respectively, were located in the outer membrane region (Figure 2). As shown in Figure 2A and C, 47 and 69% of the proteins were not predicted or annotated with clear subcellular location by PSORTb and UniProt. It’s noteworthy that UniProt annotated 26 membrane proteins and 45 TM proteins, some of which may also be OMPs. To balance the prediction coverage and confidence of prediction, the 101 proteins (Supplementary Table 1) that were predicted as OMPs by at least two servers were selected for further analysis.

### Homology and Adhesion of *V. parahaemolyticus* and *Vibrio* spp. Proteins

The sequence homology of 101 OMPs identified in *V. parahaemolyticus* RIMD 2210633 with heterogenous serotypes and isolates of *V*. *parahaemolyticus* was analyzed by BLAST in NCBI. The results showed that these OMPs are conserved (>98%) in *V*. *parahaemolyticus*. The BLAST of the identified OMPs with 32 other *Vibrio* species, seven closely related marine bacteria, and six other common bacteria showed that 70% of the OMPs (e.g., previously reported OmpA, OmpW, and OmpU) had relatively high but uneven sequence homology with other *Vibrio* species and marine bacteria, such as *Photobacterium*, *Shewanella*, *Aliivibrio*, *Salinivibrio*, *Enterovibrio*, and *Grimontia*. Interestingly, most of the OMPs were found to share a high sequence homology with nine *Vibrio* species, including *V. alginolyticus*, *V. diabolicus*, *V. antiquaries*, *V. campbelli*, *V. harveyi*, *V. owensii*, *V. rotiferianus*, *V. jasicida*, and *V. natriegens*, which indicated a close genetic relationship between these strains. Notably, *V. diabolicus* and *V. antiquaries* are isolated from a deep-sea hydrothermal vent environment, and their prevalence and pathogenicity in coastal systems and seafood samples remains unclear (Turner et al., 2018).

However, we did identify 19 OMPs that exhibited relatively good specificity for *V. parahaemolyticus*. As listed in Table 1, six OMPs, including OmpA-like domain-containing protein (VPA0731), outer membrane protein (VPA0316), AraC/XylS family transcription factor (VP0854), and three hypothetical proteins (VP2191, VPA1456, and VPA0548), shared negligible homology (<32%) with non-*Vibrio* spp. and relatively low homology (<85%) with two to nine *Vibrio* species. Eleven OMPs shared a low homology (38 – 74%) with several non-*Vibrio* strains, six of which (NP_799595.1, NP_800886.1, NP_799735.1, and NP_797735.1, CsuD protein, and MtrC) only had a high homology (83 – 99%) with one to three *Vibrio* species (*V. alginolyticus*, *V. diabolicus*, and *V.antiquaries*). The last five OMPs (flagellin, OmpA, NP_799317.1, NP_799752.1, and NP_800976.1) shared a sequence homology of less than 89% with the closely related nine *Vibrio* species. There are two differential OMPs that are not listed in Table 1. The long-chain fatty acid transport protein (NP_798592.1) was only found to display a high homology (93%) with *V. alginolyticus*, *V. diabolicus*, *V. antiquaries*, and *V. rotiferianus* and shares a 66% identify with *Photobacterium* and *Shewanella*. The outer membrane porin protein (NP_797387.1) is specific to the *V. parahaemolyticus*, *V. diabolicus*, *V. antiquaries*, *V. campbelli*, *V. harveyi*, and *V. jasicida* and was not found in the non-*Vibrio* spp. strains.

**TABLE 1:** The homology of differential OMPs of *V. parahaemolyticus* identified by bioinformatic tools.

**Accession**	**Description**	**α -Helix**	**Adhesion**	**Sequence homology**		
				***V. parahaemolyticus***	**32 Species of *Vibrio* spp.**	**non-*Vibrio* strains**
NP_800241.1	OmpA-like domain-containing protein	0	0.200	92% 100	74%, *V. harveyi*	*N*
					74%, *V. diabolicus*	
NP_798570.1	hypothetical protein	1	0.125	91% 280	<66%, nine species^a^	*N*
NP_800966.1	hypothetical protein	1	0.103	97% 430	<75%, nine species	*N*
NP_799826.1	Outer membrane protein	0	0.686	92% 70	<76%, nine species	*N*
NP_797233.1	AraC/XylS family transcription factor	1	0.250	99% 500	<81%, nine species	*N*
NP_800058.1	hypothetical protein	0	0.353	98% 73	<85%, nine species	*N*
NP_799595.1	hypothetical protein	0	0.325	99% 54	99%, *V. diabolicus*	*Photobacterium* 50%
NP_800886.1	hypothetical protein	0	0.586	97% 20	83%, *V. cholerae*	*Shewanella et al*. 57%
					97%, *V. mimicus*	
NP_799735.1	Putative efflux pump channel protein	0	0.565	97% 344	97%, *V. alginolyticus*	*Shewanella et al*. 55%
					80%, *V. natriegens*	
NP_797735.1	hypothetical protein	0	0.468	95% 287	87%, *V. natriegens*	*Photobacterium* 74%
NP_801014.1	CsuD protein	0	0.411	97% 444	87%, *V. alginolyticus*, *V. diabolicus, V. antiquaries*	*Enterovibrio* 60%
NP_797599.1	MtrC	0	0.430	97% 467	*96%, V. diabolicus, V. antiquaries*	*Enterovibrio* 52%
OAR40234.1	flagellin	0	0.393	98% 304	<81%, nine species	*Grimontia et al*. 47%
NP_797143.1	OmpA	0	0.311	97% 78	<88%, nine species	*Photobacterium* 50%
NP_799317.1	vitamin B12 receptor	0	0.608	97% 154	<89%, nine species	*Aeromonas et al*. 41%
NP_799752.1	OMP_b-brl domain-containing protein	0	0.611	95% 122	<89%, nine species	*Grimontia* 67%
NP_800976.1	TonB system receptor	0	0.508	97% 405	<89%, nine species	*Aliivibrio et al*. 59%

*^*a*^homology was only found with the nine species including *V. alginolyticus*, *V. diabolicus*, *V. antiquaries*, *V. campbelli*, *V. harveyi*, *V. owensii, V. rotiferianus*, *V. jasicida*, and *V. natriegens*.*

Table 2 shows that there were 15 OMPs that shared at least a 65% sequence homology across 23 or more species out of the 32 *Vibrio* species. These proteins are widely conserved in *Vibrio* spp. because they are involved in the fundamental structure or biological function, such as the flagellar motor protein complex (e.g., flgH1 and motY), new OMPs assembly (e.g., BamA), polysaccharide assembly (e.g., LptD), fimbriae assembly (NP_799125.1 and NP_796512.1), the TolC subfamily within the efflux systems (NP_798013.1, NP_798092.1, and NP_796804.1), maintaining the outer membrane integrity (VacJ), polysaccharide binding, and carbohydrate metabolic process (NP_797134.1). While the protective efficacy of proteins OmpW (Mao et al., 2007), OmpK (Li et al., 2010b), LptD (Zha et al., 2016), and Pal (Li et al., 2010a) has already been studied. The other OMPs that are conserved in more *Vibrio* species were newly identified in this study.

**TABLE 2:** The homology of differential OMPs of *Vibrio* spp. identified by bioinformatic tools.

**Accession**	**Description**	**α -Helix**	**Adhesion**	**Sequence homology**		
				***V. parahaemolyticus***	**32 Species of *Vibrio* spp.**	**non-*Vibrio* strains^a^**
NP_798689.1	Outer membrane protein assembly factor YaeT BamA	0	0.439	98% 63	>70%, 31 species	53–78%
NP_796804.1	Outer membrane channel protein Tolc	0	0.427	91% 62	>70%, 31 species	51–69%
NP_799125.1	Fimbrial assembly protein	0	0.222	93% 106	>65%, 31 species	52–70%
NP_796512.1	General secretion pathway protein D	0	0.142	93% 49	>75%, 31 species	54–70%
NP_797161.1	Flagellar basal body L-ring protein	0	0.681	99% 33	>70%, 30 species	*Salinivibrio* 58%
NP_798092.1	Hypothetical protein	0	0.336	93% 160	>65%, 29 species	52–62%
NP_798593.1	VacJ lipoprotein	0	0.289	99% 99	>65%, 29 species	50–62%
NP_796718.1	LPS-assembly protein LptD	0	0.649	92%117	>60%, 29 species	44–48%
NP_798013.1	Agglutination protein tolC super family	0	0.411	99% 94	>70%, 28 species	51–68%
NP_798490.1	Sodium-type flagellar protein MotY	0	0.255	94% 60	>70%, 28 species	62–65%
SUQ25448.1	Outer membrane protein OmpK	0	0.763	79% 43	>65%, 27 species	65–80%
NP_797646.1	Lipoprotein	1	0.416	97%26	>70%, 25 species	40–70%
NP_797440.1	Peptidoglycan-associated lipoprotein Pal	0	0.333	99%24	>70%, 25 species	61–64%
NP_797134.1	Chitobiase	0	0.386	99% 380	>70%, 24 species	57–74%
NP_799606.1	Outer membrane protein OmpW	0	0.774	90%51	>65%, 23 species	*Grimontia* 71%

*^*a*^These non-*Vibrio* strains include *Aliivibrio* Sp., *Salinivibrio* Sp., *Enterovibrio* Sp.,*Grimontia* Sp., and *Photobacterium* Sp.*

**FIGURE 3:**
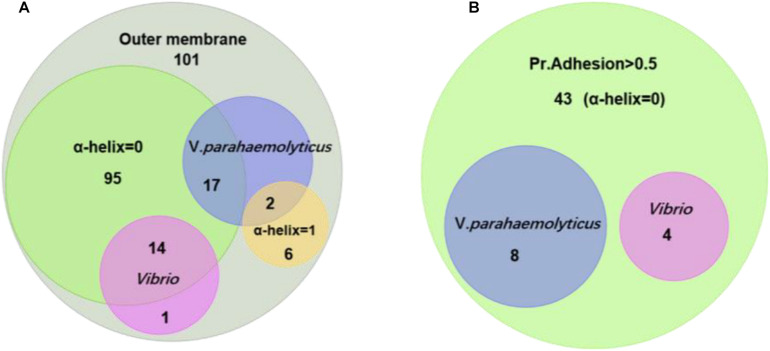
Homology and adhesion. **(A)** The distribution and crossover of 101 outer membrane protein candidates. There are 15 proteins that are homologous in *Vibrio*; 19 proteins that are homologous in *V. parahaemolyticus*; 95 proteins that have no TM α-helix; and six proteins that have one TM α-helix. **(B)** There are 43 proteins that have a Pr. adhesion score of more than 0.5; four proteins are homologous in *Vibrio*; and eight proteins are homologous in *V. parahaemolyticus*.

Figure 3A shows the relationship of the identified OMPs with the number of α-helix structure, *V. parahaemolyticus* differential OMPs, *Vibrio* spp. conserved OMPs. Most candidate OMPs had no TM α-helix, which indicated a dominant β-barrel structure. The analysis of protein antigenicity by VaxiJen showed that 96 out of the 101 proteins exhibited a higher score than the cut-off (>0.4), which indicated the identified OMPs were potentially antigenic proteins. Adhesion proteins help bacteria attach to the host cell receptors and promote colonization. Creating a vaccine and generating associated neutralizing antibodies against adhesion proteins is effective in preventing bacterial infections at an early stage (Wizemann et al., 1999). The results of the analysis by Vaxign showed that 43 OMPs had adhesion probability higher than 0.5 and were predicated to be related to adhesion (Figure 3B). Among these, eight OMPs (outer membrane protein NP_799826.1, hypothetical protein NP_800886.1, putative efflux pump channel protein NP_799735.1, vitamin B12 receptor NP_799317.1, OMP_b-brl domain-containing protein NP_799752.1, and TonB system receptor NP_800976.1) were more specific to *V. parahaemolyticus* (Table 1) and four OMPs (flgH, LptD, OmpK, and OmpW) were widely conserved in *Vibrio* spp. (Table 2).

### Surface Proteins Identified by Enzymic Shaving and Mass Spectrometry

Trypsin was specific to hydrolyze carboxyl side of lysine and arginine residues in polypeptide chain and was widely used in proteomic studies (Olaya-Abril et al., 2014). To experimentally study the OMPs on the cell surface, trypsin was first used to enzymatically shave off the intact cells. The results of LC-MS/MS showed although little intracellular proteins were observed for the control sample without trypsin, there were no known OMPs identified in the experimental group after 30 min shaving except some intracellular proteins (Table 3). The identified BamD that annotated as OMP actually faced the periplasm side of the outer membrane (Han et al., 2016). *Vibrio* was known to express the capsule (K antigen) on the cell to adapt the environment and escape the phagocytosis (Pettis and Mukerji, 2020). This may limit the availability of OMPs to trypsin digestion. Many studies have shown that the capsule or lipopolysaccharides consist of α-1,3, α-1,2, α-1,4, β-1,4, β-1,3, α-1,5, β-1,5, α-1,6, and β-1,6 glycosidic bond (Chen et al., 2007). The endoglycosidases were known to cleavage the specific glycosidic bond inside the polysaccharide and have been reported to reduce the capsule level of *Streptococcus pneumoniae* (Middleton et al., 2018). With the combination of four endoglycosidase and one exoglycosidase in a one-step enzymatical digestion of the intact cells, the sugar content in the supernatant increased from 4.89 to 12.90 mg/100 g after 6 h (Supplementary Figure 1). This indicated the reduction of exopolysaccharides on the cell. The following enzymatical shaving of the intact cells by trypsin showed more intracellular proteins in both the positive and control group were observed, but peptides of some proteins were mainly observed in the experimental group and further analysis with UniProt confirmed there were 14 OMPs identified. These OMPs were OmpA (VPA1186, VPA0248, and VP0764), Omp (VPA0166), OmpU (VP2467), BamA (VP2310), TolC (VP0425), GspD (VP0133), OmpK (VP2362), lpp (VPA1469), Pal (VP1061), 5′-nucleotidase (VP0748), agglutination protein (VP1634), and putative iron (III) compound receptor (VPA1435).

**TABLE 3:** The identified OMPs in *V. parahaemolyticus* O3:K6 (RIMD 2210633) by proteomic analysis.

**Accession**	**Description**	**Function**	**Mw (kD)**	**Peptide (−^a^)**	**Peptide (T^b^)**	**Peptide (G^c^)**	**Peptide (G + T^d^)**	**Bioinformatics**
NP_797127.1	5′-nucleotidase	Degradation for nutrition	62.175	−	−	1	1	+
NP_798741.1	OmpK	Receptor for vibriophage	29.877	−	−	−	2	+
NP_800979.1	Outer membrane lipoprotein (lpp)	Distance controls of membranes	8.671	−	−	−	1	+
NP_800945.1	Putative iron (III) compound receptor	Siderophore uptake transporter	77.059	−	−	−	1	+
NP_800696.1	OmpA	Porin activity	36.014	−	−	−	5	+
NP_799758.1	OmpA	Porin activity	35.553	−	−	−	9	+
NP_799676.1	Putative Omp	Porin activity	37.974	−	−	1	4	+
NP_798846.1	OmpU	Passive diffusion of small molecules	36.285	−	−	−	10	+
NP_798689.1	BamA	Omp assembly complex	90.053	−	−	−	3	+
NP_798013.1	Agglutination protein	Efflux transmembrane transporter	48.69	−	−	−	1	+
NP_797440.1	Pal	Division and cell integrity	18.713	−	−	1	3	+
NP_797143.1	OmpA	Porin activity	34.073	−	−	2	9	+
NP_796804.1	TolC	Efflux transmembrane transporter	47.983	−	−	−	8	+
NP_796512.1	GspD	Protein secretion by the T2S	73.317	−	−	−	1	+

*^*a*^Control group treated with only the shaving buffer of trypsin (1× PBS, 20% [w/v] sucrose, 10 mM DTT).*

*^*b*^Experimental group treated with trypsin in shaving buffer.*

*^*c*^Experimental group treated with glycosidases in phosphate buffer (pH 5.5) and the shaving buffer of trypsin.*

*^*d*^Experimental group treated with glycosidases in phosphate buffer (pH 5.5) and trypsin in shaving buffer.*

### Protein Structure of the Omps Identified by Both Reverse Vaccinology and Surface Proteome

**FIGURE 4:**
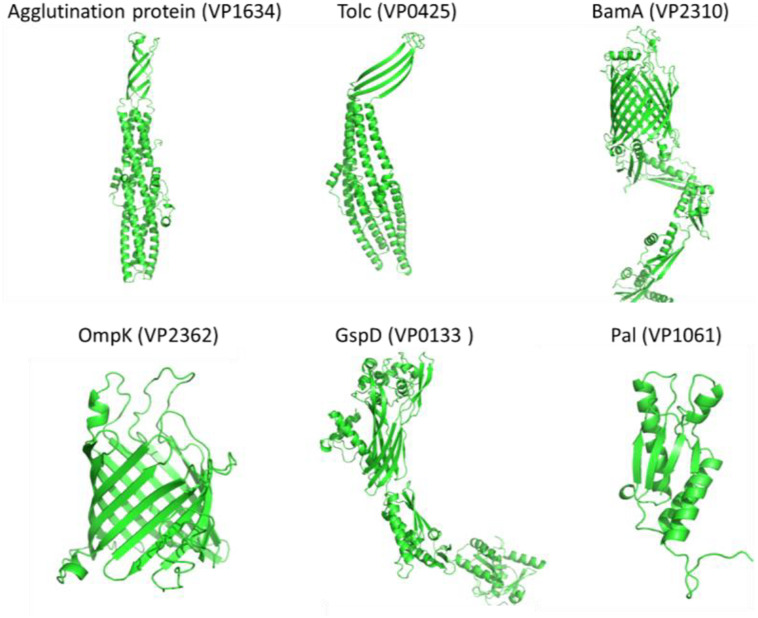
The simulated protein structures of six widely conserved *Vibrio* Omps (Agglutination protein, Tolc, BamA, OmpK, GspD, and Pal) identified by both reverse vaccinology and surface proteomic analysis.

The results in Tables 2, 3 revealed that OmpK, agglutination protein, Pal, TolC, GspD, and BamA were identified by the bioinformatical screening of the encoded protein database and enzymatical shaving the intact cells of *V. parahaemolyticus*. Protein simulation by I-TASSER and TrRosetta (Figure 4) showed that all these protein structures had an estimated TM score ≥0.5 and C-score more than −1.5. The tolC super family protein agglutination protein (VP1634) and Tolc (VP0425) had a typical β-barrel domain on the outer membrane and α-helical colied coli structure protrude deep into the periplasm (Zgurskaya et al., 2011). The GspD belong to the type II secretory system also had a similar structure which promote the secretion of protein. The BamA and OmpK structure had a common β-barrel structure of a porin protein, which are involved in protein assembly and receptor.

## Discussion

The novel OMPs as immunogens for preparing diagnostic antibodies and subunit vaccine against *V. parahaemolyticus* and *Vibrio* spp. are of interest in aquaculture and food safety. Traditional study of the major OMPs was unsystematic and time consuming. Based on bioinformatic tools with different filter thresholds and applications, reverse vaccinology processes a large quantity of genetic or proteomic data and minimize the targets in the following *in vivo* experiments. The surface proteome based on enzymatical shaving of the extracellular peptides of the cells and massive identification of the peptides provides an experimental insight of the surface available OMPs. The two comprehensive and complementary strategies were both adopted in this work to study the diagnostic surface antigens of *V. parahaemolyticus* and *Vibrio* species.

For the bioinformatic analysis, signal sequences presented at the N-terminal were first predicted because most bacterial OMPs have an N-terminal signal sequence that promotes protein secretion (Gao et al., 2016). Our result identified three different signal peptides. The Sec signal peptide directs unfolded protein translocation across the plasma membrane in prokaryotes (Tsirigotaki et al., 2017). TAT signal peptide actively translocate the folded proteins across the lipid bilayer of the membrane (Armenteros et al., 2019) and are generally longer and less hydrophobic (Berks, 2015). The lipoprotein signal peptides play an important role in the maturation of bacterial lipoprotein (Kitamura and Wolan, 2018), many of which are surface-exposed (Wilson and Bernstein, 2016). To cross-check the subcellular location of candidate proteins, three parallel prediction tools were used in this study. Among them, PSORTb was reported to be the most precise bacterial localization prediction tool available based on a report in 2010 (Yu et al., 2010). The results in Figure 2D showed that when CELLO and PSORTb, CELLO and UniProt, PSORTb and UniProt were used to cross-check, 86, 53, and 54 proteins were, respectively, predicted as OMPs. Parallel use of the three prediction tools and retaining the proteins predicted by two or three methods obtained more candidate OMPs (101) with better coverage and accuracy.

The homology analysis of the *V. parahaemolyticus* OMP candidates by BLASTp revealed a relatively conserved and complex genetic relationship with the other *Vibrio* species and some marine bacteria, which provide the insight of possible cross-reaction or cross-protection with these OMPs. Based on the overall homology analysis, 15 OMPs that are broadly conserved in the genus of *Vibrio* were identified. Four of them were predicted to be adhesion proteins and were potentially multivalent subunit vaccine candidates against vibriosis. Many of these conserved candidate proteins are involved in some significant function. For example, BamA participates in the delivery of extracellular membrane proteins and is the core component of the β-barrel assembly machinery BAM complex (Singh et al., 2017). Peptidoglycan-associated lipoprotein (Pal) stabilizes the outer membrane by providing a non-covalent biding through the peptidoglycan layer (Parsons et al., 2006). The general secretion pathway protein (GspD) in *V. cholerae* directly involves the production of rugose polysaccharide and the secretion of cholera toxin and hemolysin (Ali et al., 2000). The TolC family protein provides a channel for the cell to connect with the external environment during export and plays a role in the bacterial efflux pumps (Koronakis et al., 2000). The protein LptD is responsible for lipopolysaccharide transport and insertion into the outer membrane (Dong et al., 2014). The flagellar basal body L-ring protein (flgH), sodium-type flagellar protein motY, and fimbrial assembly protein involved in motility and adhesion (Rehman et al., 2019). The OmpK and OmpW are porin proteins that are involved in the transport of small hydrophobic molecules across the bacterial outer membrane (Hong et al., 2006).

Due to the complex homology, completely specific *V. parahaemolyticus* OMPs were not found, but 19 differential OMPs were obtained in this study. The CsuD protein and MtrC (Beliaev et al., 2001), respectively, has fimbrial usher porin activity and terminal Fe(III) reductase activity. Vitamin B12 receptor and TonB system receptor play a role in nutrition uptake and transportation (Köster, 2001). OmpA and OmpA like protein function as an adhesin and invasin, participate in biofilm formation, and serve as a bacteriophage receptor (Smith et al., 2007). The AraC-XylS family proteins are known as transcriptional proteins regulator (Tobes and Ramos, 2002). Further analysis of the structure, topology, and sequence homology of these OMPs may reveal the specific extracellular epitopes for *V. parahaemolyticus* and overcome the challenge of discovering diagnostic targets.

Previous immunoproteomic work performed by Li et al. (2010a) identified the putative iron (III) compound receptor (VPA1435), TolC (VP0425), and OmpA (VPA0764), conserved hypothetical protein (VP2850), BamA (VP2310), and Pal (VP1061) as the immunogenic OMPs of *V. parahaemolyticus*. Similar work described by another group identified the lipopolysaccharide-assembly protein, LptD, hypothetical protein (VP0802), Maltoporin (LamB), OmpA, OmpU, and OmpK, as well as hypothetical VP1243 and VP0966 (Li et al., 2014). Recently, Yonekita et al. (2020) reported that a diagnostic antibody prepared against a whole cell antigen was specific to 12 serotypes of *V. parahaemolyticus* and did not react with the other six *Vibrio* species. The antigen was identified to be outer membrane lipoprotein (NP_800979.1 and VPA1469). All of these proteins were predicted as OMPs in this study, except VP2850, VP1243, and VP0966, which were only predicted by CELLO. Importantly, we confirmed the homology of these OMPs among the 32 species of *Vibrio* and closely related non-*Vibrio* strains at the sequence level. LptD, Pal, BamA, TolC, OmpK, and OmpW (Table 2) were conserved in at least 23 *Vibrio* species. The lipo protein VPA1469 was also found to have a high homology with *V. campbelli*, *V. harveyi V. owensii*, and *V. rotiferianus*. Apart from these Omps, we identified new OMPs that were conserved in *Vibrio* spp. or relatively specific to *V. parahaemolyticus* for development of better diagnostic tools and vaccines.

In our surface proteome study, direct shaving of PFA fixed *V. parahaemolyticus* cells resulted in no OMPs but some cytoplasmic proteins, which were also found in many previous surface proteome studies of Gram-negative strains (Fagerquist and Zaragoza, 2018). This is mainly because the soft cell membrane is more vulnerable to osmotic lysis (Olaya-Abril et al., 2014). Combination of four endoglycosidases and an exoglycosidase in a one-step digestion reduced capsule level of *V. parahaemolyticus* in this study. The following shaving with trypsin and analysis indicated 14 OMPs, also identified in the reverse vaccinology strategy, may have a better accessibility on the cell surface. Rodríguez-Ortega found the number of surface-exposed proteins for group A *Streptococcus* varied from strain to strain and it’s mostly because of the different capsule content (Rodríguez-Ortega et al., 2006). Our results also suggested a steric hindrance effect of the capsular polysaccharide, which was widely expressed by *Vibrio* species, most likely masked the OMPs from enzymatical shaving of the intact cell. The mask of many OMPs by capsule may also limit its reaction with antibodies, which should be considered in immunodetection and vaccine development. The expression of capsule of *Vibrio* was regulated by temperature, divalent cation (Ca^2+^ and Mn^2+^), oxygen content, and host environment (Pettis and Mukerji, 2020). Further study of the surface proteome of *V. parahaemolyticus* with minimal capsule expression will elucidate more stably expressed OMPs and the optimal enrich broth for the immunodetection of *V. parahaemolyticus* from various environmental samples.

In short, reverse vaccinology analysis of the surface proteins based on the protein database of *V. parahaemolyticus* in NCBI comprehensively predicted 101 OMPs and revealed 19 differential OMPs of *V. parahaemolyticus* and 15 conserved OMPs of *Vibrio* spp. Surface proteome study of *V. parahaemolyticus* by enzymatical shaving of the exopolysaccharide and proteins on the intact cell identified 14 OMPs, which mainly belong to the conserved OMPs of *Vibrio* spp. Future study of the capsule expression level, surface proteome of *V. parahaemolyticus* under various environments, and the interaction of *V. parahaemolyticus* with antibodies against these Omps will further reveal the diagnostic surface antigen or epitopes of *V. parahaemolyticus* and *Vibrio* spp.

## Data Availability Statement

The datasets presented in this study can be found in online repositories. The names of the repository/repositories and accession number(s) can be found in the article/ Supplementary Material.

## Ethics Statement

This article does not contain any studies with human participants or animal experiments.

## Author Contributions

WW and SP initiated the study. JL and WW mainly conducted the experiment, analyzed the data, and wrote the manuscript. JL, LL, SG, and QY participated the data analysis. LG revised the manuscript. All the authors read and approved the manuscript.

## Conflict of Interest

The authors declare that the research was conducted in the absence of any commercial or financial relationships that could be construed as a potential conflict of interest.

## Footnotes

1 https://blast.ncbi.nlm.nih.gov/Blast.cgi
